# Programmed death-ligand 1 expression in gastric adenocarcinoma is a poor prognostic factor in a high CD8+ tumor infiltrating lymphocytes group

**DOI:** 10.18632/oncotarget.12603

**Published:** 2016-10-12

**Authors:** Hyeyoon Chang, Woon Yong Jung, Youngran Kang, Hyunjoo Lee, Aeree Kim, Han Kyeom Kim, Bong Kyung Shin, Baek-hui Kim

**Affiliations:** ^1^ Department of Pathology, Korea University Guro Hospital, Korea University College of Medicine, Guro-gu, Seoul, Republic of Korea; ^2^ Department of Pathology, Catholic Kwandong University International St Mary's Hospital, Incheon, Republic of Korea; ^3^ Department of Pathology, Green Cross Laboratories, Yongin, Kyeonggi-Do, Republic of Korea; ^4^ Department of Pathology, Kangbuk Samsung Hospital, Sungkyunkwan University College of Medicine, Seoul, Republic of Korea

**Keywords:** gastric adenocarcinoma, PD-1, PD-L1, CTLA-4, prognosis

## Abstract

Gastric adenocarcinoma is one of the most common causes of cancer-related death. In this study, we conducted immunohistochemical studies for PD-L1, PD-1, CTLA-4, and CD8 using tissue microarrays from 464 gastric cancer samples and evaluated the correlations between their expression, clinicopathologic factors, and five-year overall survival. PD-L1 and PD-1 expression was significantly correlated with several adverse prognostic pathologic factors, including higher T-stage, diffuse Lauren histologic type, and lymphatic invasion. Conversely, CTLA-4 expression was correlated with factors of favorable clinical outcomes. A complete case analysis revealed that high PD-L1 and PD-1 expression had an adverse effect on five-year overall survival in univariate analyses. Subgroup analyses wherein patients were divided into two groups according to CD8+ tumor infiltrating lymphocyte levels (high and low) showed that high PD-L1 expression was a significant adverse prognostic factor only in the high CD8+ tumor-infiltrating lymphocytes group. Further research and clinical trials are needed to determine the clinical usefulness of these findings.

## INTRODUCTION

Gastric adenocarcinoma is one of the most common causes of cancer-related death, especially in Eastern Asia, Central and Eastern Europe, and South America. [1] Although the incidence of gastric adenocarcinoma has declined dramatically, gastric cancer is the second most common cause of cancer death worldwide [2].

Cytotoxic T-lymphocyte-associated antigen 4 (CTLA-4; also known as CD152) is expressed on T-cells and is known to act as an inhibitory receptor competing with the T-cell costimulatory receptor, CD28. CTLA-4 shares the same ligands, CD80 and CD86, which are expressed on antigen-presenting cells (APCs), and blocks the early stages of T cell activation as a result [3]. CTLA-4 is also involved in tumor immunity associated with negative regulation of T-cell receptors [4]. Additionally, constitutive CTLA-4 expression in various human malignant solid tumor cell lines enabled not only its role in negative control of immune responses but also in the neoplastic process [5].

Programmed cell death protein 1 (PD-1; also known as CD279), a member of the B7-CD28 family, is another key inhibitory receptor expressed on activated T-cells, particularly cytotoxic T-cells, B-cells, monocytes, and natural killer cells, and harbors two ligands, programmed death-ligand 1 (PD-L1) (B7-H1, CD274) and PD-L2 (B7-DC) [6]. Among these, PD-L1 expressed in tumor cells or APCs binds to PD-1 receptors on activated T-cells and inhibits T-cell responses by inhibiting antigen receptor signaling [7]. The PD-1/PD-L1 pathway is thought to be the key regulator of tumor-induced immune suppression [8]. Expression of PD-L1 is reported to be associated with poor prognosis in various tumors [9–11].

The purpose of cancer immunotherapy research is the development of methods to enhance existing host anti-tumor responses. Cancer immunotherapy, by blocking inhibitory receptors such as CTLA-4 and PD-1/PD-L1, is being actively studied and applied to the treatment of various cancers, including melanoma, and lung and kidney cancers [12].

The Cancer Genome Atlas Research Network defined four molecular subtypes of gastric adenocarcinomas: tumors positive for Epstein–Barr virus, microsatellite unstable tumors, genomically stable tumors, and tumors with chromosomal instability [13]. Tumors positive for Epstein–Barr virus showed elevated PD-L1 expression, suggesting the rolls of immune checkpoint inhibitors in gastric cancer.

In this study, we conducted immunohistochemical (IHC) studies of CTLA-4, PD-1, PD-L1, and CD8 using 464 gastric cancer tissue microarrays and evaluated the expression of molecular markers related to immune checkpoint inhibition. We also analyzed the correlations between molecular-marker expression, clinicopathologic factors, and survival outcomes.

## RESULTS

### Expression of molecular markers and correlation with clinicopathological features

**Figure 1:**
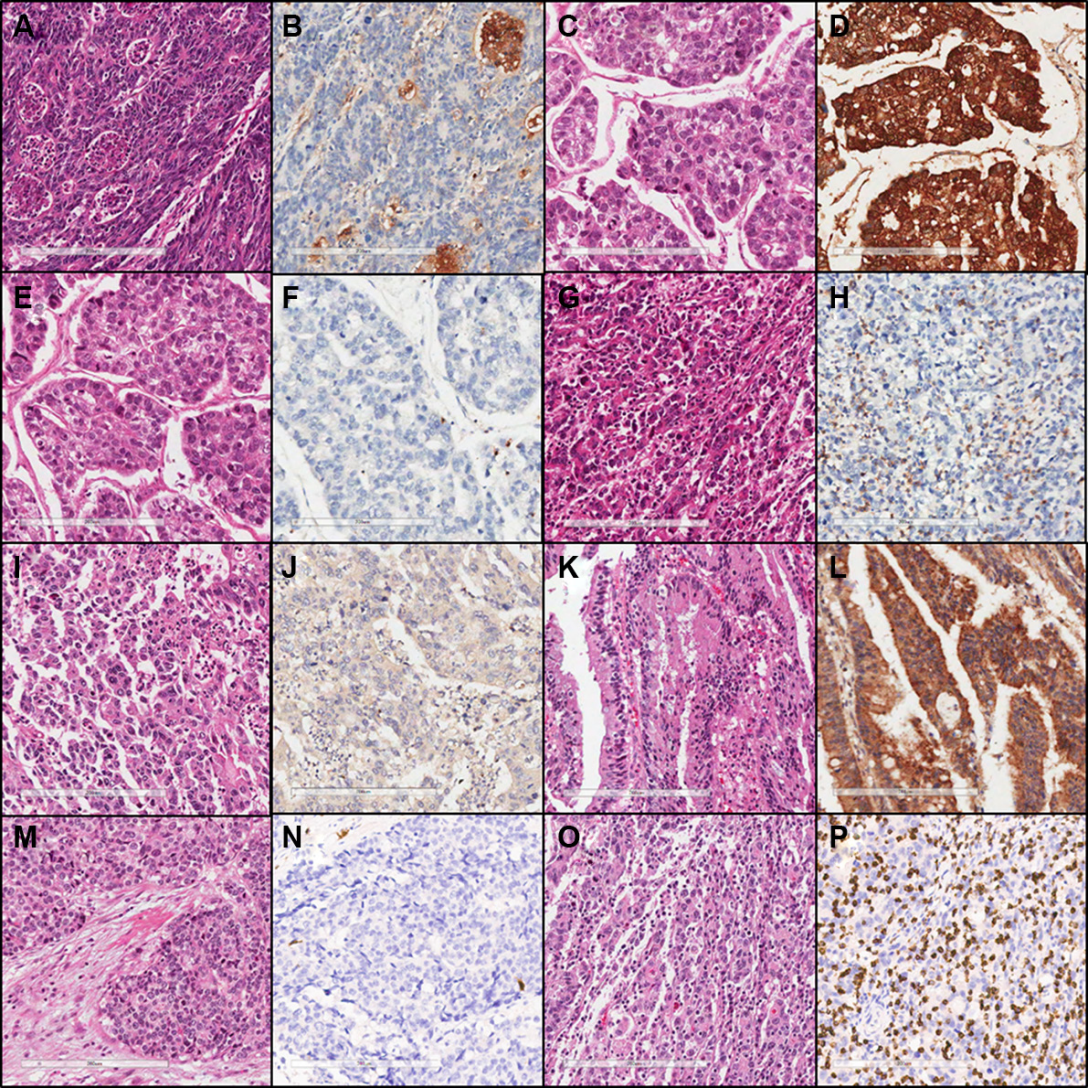
Representative microscopic images of hematoxylin & eosin and immunohistochemical staining (×200) of low PD-L1 (A, B), high PD-L1 (C, D), low PD-1 (E, F), high PD-1 (G, H), low CTLA-4 (I, J), high CTLA-4 (K, L), low CD8 (M, N), and high CD8 expression (O, P)

In total, representative tumor sections from 464 gastric adenocarcinoma tissues were constructed from FFPE tissue, but thirteen cases were excluded because of loss of tissue cores during cutting, mounting, or the IHC staining process. The representative microscopic images from IHC staining are shown in Figure 1. Clinicopathologic characteristics of patients in relation to molecular markers are shown in Table 1.

**Table 1:** Associations of PD-L1, PD-1, CD8, and CTLA-4 expression levels with clinicopathological factors

	PD-L1 (%)			PD-1 (%)			CD8 (%)			CTLA-4 (%)		
	Low	High	*p*-value	Low	High	*p*-value	Low	High	*p* -value	Low	High	*p*-value
Age (yr.)			0. 453			0. 429			0. 489			0. 840
≤ 65	94 (68. 12)	201 (64. 22)		143 (63. 56)	152 (67. 26)		151 (67. 11)	144 (63. 72)		174 (64. 93)	121 (66. 12)	
> 65	44 (31. 88)	112 (35. 78)		82 (36. 44)	74 (32. 74)		74 (32. 89)	82 (36. 28)		94 (35. 07)	62 (33. 88)	
T-stage			< 0. 001*			0. 021^*^			0. 225			0. 031^*^
Tis, T1	108 (78. 26)	65 (20. 77)		101 (44. 89)	72 (31. 86)		96 (42. 67)	77 (34. 07)		96 (35. 82)	77 (42. 08)	
T2	11 (7. 97)	51 (16. 29)		24 (10. 67)	38 (16. 81)		26 (11. 56)	36 (15. 93)		30 (11. 19)	32 (17. 49)	
T3	6 (4. 35)	60 (19. 17)		33 (14. 67)	33 (14. 60)		33 (14. 67)	33 (14. 6)		40 (14. 93)	26 (14. 21)	
T4	13 (9. 42)	137 (43. 77)		67 (29. 78)	83 (36. 73)		70 (31. 11)	80 (35. 4)		102 (38. 06)	48 (26. 23)	
Tumor grade			< 0. 001^*^			< 0. 001^*^			< 0. 001^*^			0. 003^*^
WD	51 (36. 96)	20 (6. 39)		50 (22. 22)	21 (9. 29)		49 (21. 78)	22 (9. 73)		31 (11. 57)	40 (21. 86)	
MD	66 (47. 83)	96 (30. 67)		92 (40. 89)	70 (30. 97)		96 (42. 67)	66 (29. 2)		93 (34. 70)	69 (37. 70)	
PD	21 (15. 22)	197 (62. 94)		83 (36. 89)	135 (59. 73)		80 (35. 56)	138 (61. 06)		144 (53. 73)	74 (40. 44)	
Lauren histologic type			< 0. 001^*^			0. 001*			0. 001^*^			0. 034^*^
Intestinal	130 (94. 20)	166 (53. 04)		164 (72. 89)	132 (58. 41)		46 (42. 99)	132 (58. 41)		165 (61. 57)	131 (71. 58)	
Diffuse, mixed, and indeterminate	8 (5. 80)	147 (46. 96)		61 (27. 11)	94 (41. 59)		61 (57. 01)	94 (41. 59)		103 (38. 43)	52 (28. 42)	
Lymph-node metastasis			< 0. 001^*^			0. 221			0. 638			0. 250
NO	114 (82. 61)	117 (37. 38)		122 (54. 22)	109 (48. 23)		118 (52. 44)	113 (50)		131 (48. 88)	100 (54. 64)	
N1 to N3	24 (17. 39)	196 (62. 62)		103 (45. 78)	117 (51. 77)		107 (47. 56)	113 (50)		137 (51. 12)	83 (45. 36)	
Lymphatic invasion			< 0. 001^*^			0. 013^*^			0. 486			0. 479
Negative	117 (84. 78)	182 (58. 15)		162 (72)	137 (60. 62)		153 (68)	146 (64. 6)		174 (64. 93)	125 (68. 31)	
Positive	21 (15. 22)	131 (41. 85)		63 (28)	89 (39. 38)		72 (32)	80 (35. 4)		94 (35. 07)	58 (31. 69)	
Venous invasion			0. 333			0. 656			0. 828			0. 265
Negative	134 (97. 10)	296 (94. 57)		216 (96)	214 (94. 69)		214 (95. 11)	213 (95. 52)		258 (96. 27)	172 (93. 99)	
Positive	4 (2. 90)	17 (5. 43)		9 (4)	12 (5. 31)		11 (4. 89)	10 (4. 48)		10 (3. 73)	11 (6. 01)	
Perineural invasion			< 0. 001^*^			0. 089			0. 089			0. 031^*^
Negative	126 (91. 30)	204 (53. 04)		173 (76. 89)	157 (69. 47)		173 (76. 89)	157 (69. 47)		186 (69. 40)	144 (78. 69)	
Positive	12 (8. 70)	109 (34. 82)		52 (23. 11)	69 (30. 53)		52 (23. 11)	69 (30. 53)		82 (30. 60)	39 (21. 31)	

* Statistically significant *p*-values are indicated in boldface type.

Abbreviations: WD, well differentiated; MD, moderately differentiated; PD, poorly differentiated

Statistically significant correlations were found in association with PD-1, PD-L1, and CD8 expressions. CTLA-4 expression was correlated negatively with PD-L1 (*p value* = 0. 012, r = −0.118; Table 2).

**Table 2:** Intercorrelation between PD-L1, PD1, CTLA-4, and CD8+ tumor-infiltrating lymphocytes

	PD-L1			CTLA-4			CD8+ TILs		
	Low	High	*p*-value	Low	High	*p*-value	Low	High	*p*-value
PD-1			0. 034^*^			0. 376			< 0. 001^*^
Low	74	134		119	89		150	58	
High	64	179		149	94		75	168	
CD8+ TILs			0. 023^*^			0. 66			
Low	80	145		136	89				
High	58	168		132	94				
CTLA-4			0. 012^*^						
Low	70	198							
High	68	115							

* Statistically significant *p*-values are indicated in boldface type.

Abbreviations: TILs, tumor infiltrating lymphocytes.

### Prognostic value of CTLA-4, PD-L1, PD-1, and CD8 expression

**Figure 2:**
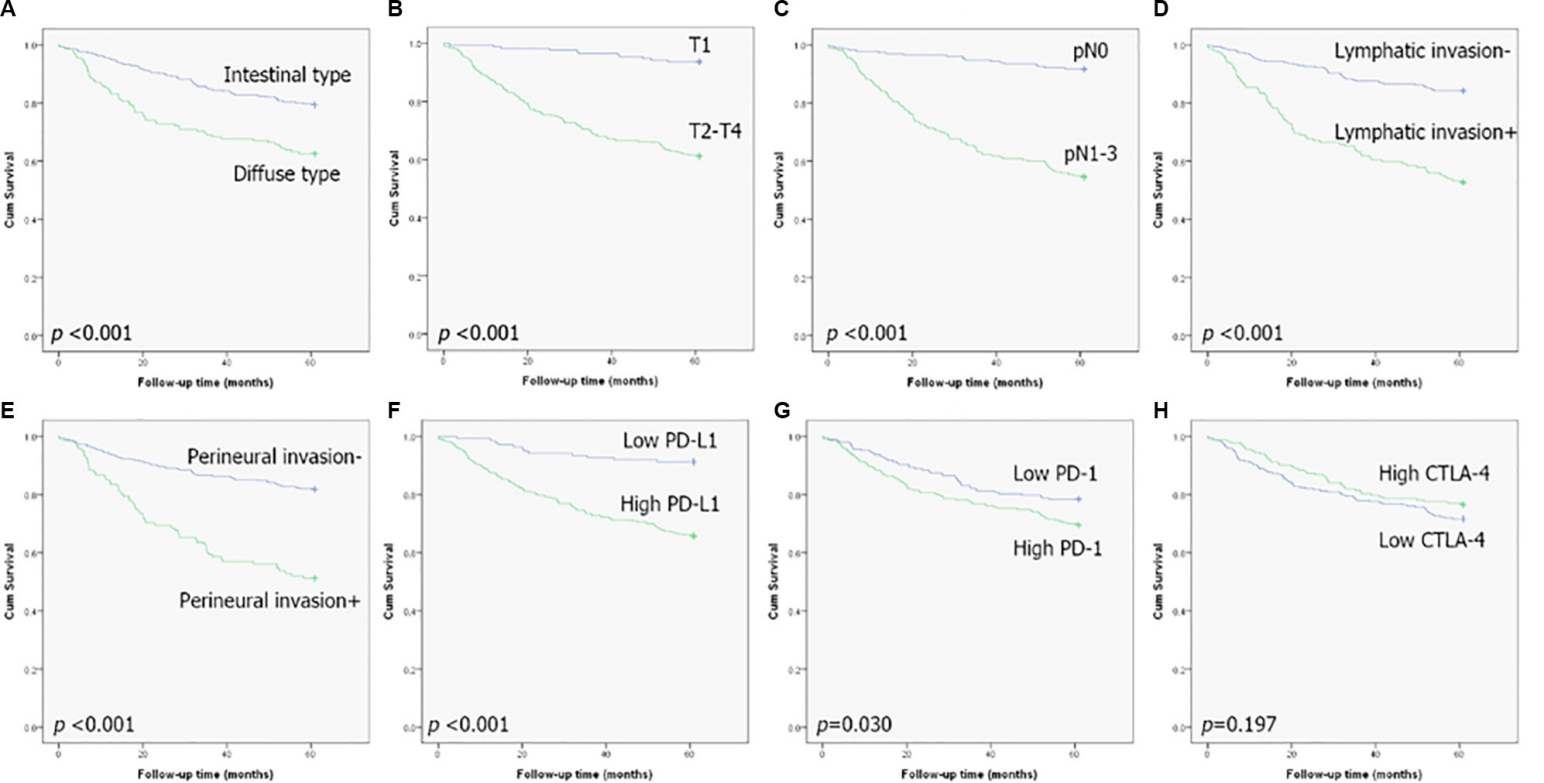
Kaplan-Meier plots of five-year survival for all patients (**A**) Lauren classification, (**B**) early versus advanced stage, (**C**) lymph-node metastasis, (**D**) lymphatic invasion, (**E**) perineural invasion, (**F**) PD-L1 expression, (**G**) PD-1 expression, and (**H**) CTLA-4 expression.

Analyzing all patients, Kaplan-Meier analyses showed that the high-expression groups for PD-L1 and PD-1 had worse overall survival (OS) at five years than low-expression groups for PD-L1 and PD-1. (*p value* < 0.001 and 0.032; Figure 2). Other pathologic factors, such as Lauren histologic grade (*p value* <0. 001), advanced gastric carcinoma (AGC) (*p value* <0. 001), lymph node metastasis (*p value* <0. 001), lymphatic invasion (*p value* < 0. 001), and perineural invasion (*p value* < 0. 001) also had statistically significant prognostic effects on five-year OS in univariate Kaplan–Meier analyses. CTLA-4 and CD8+ TILs (tumor infiltrating lymphocytes) expressions were not statistically significant.

In Cox multivariate analyses, expression of PD-L1 and PD-1 showed no significant independent prognostic effect on five-year OS (Table 3).

**Table 3:** Multivariate analyses of variables in relation to five-year overall survival of all patients

Variables	Parameter	Hazard ratio (95% CI)	*p*-value
Lauren classification	Diffuse vs. intestinal	1. 044 (0. 707–1. 544)	0. 827
Tumor stage	pT2-4 vs. pT1	0. 423 (0. 200–0. 895)	0. 024^*^
Lymph-node metastasis	Present vs. absent	0. 346 (0. 193−0. 621)	< 0. 001^*^
Lymphatic invasion	Present vs. absent	0. 581 (0. 390−0. 866)	0. 008^*^
Perineural invasion	Present vs. absent	0. 682 (0. 462−1. 005)	0. 053
PD-L1	High vs. low	0. 647 (0. 335−1. 250)	0. 195
PD1	High vs. low	1. 345 (0. 922−1. 963)	0. 124

* Statistically significant *p*-values are indicated in boldface type.

Abbreviations: CI, confidence interval.

**Figure 3:**
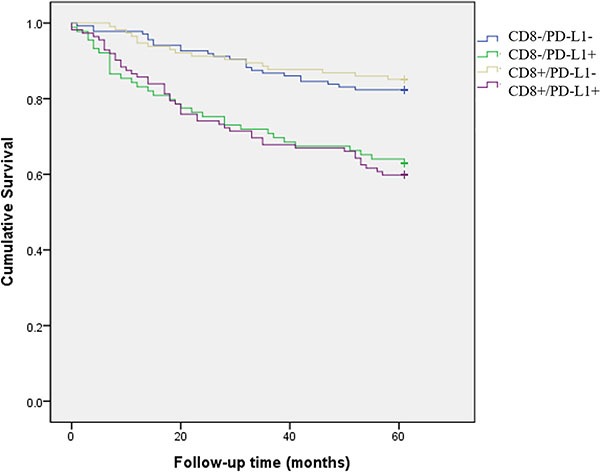
Kaplan-Meier plots of five-year survival for four patient groups: CD8-/PD-L1-, CD8-/PD-L1+, CD8+/PD-L1-, and CD8+/PD-1+ The high PD-L1 expression group (CD8-/PD-L1+, CD8+/PD-1+) showed poor survival compared to the low PD-L1 expression group (CD8-/PD-L1-, CD8+/PD-L1-).

For survival analyses to assess the combination of PD-L1 overexpression and CD8+ TILs, we classified patients into four groups: CD8-/PD-L1-, CD8-/PD-L1+, CD8+/PD-L1-, and CD8+/PD-1+ (Figure 3). Kaplan-Meier survival curves were then used to roughly separate the patients into two groups: 1) CD8-/PD-L1-, CD8+/PD-L1- and 2) CD8-/PD-L1+, CD8+/PD-1+ (*p value* < 0.001). The high PD-L1 expression group (CD8-/PD-L1+, CD8+/PD-1+) showed poor survival compared to the low PD-L1 expression group (CD8-/PD-L1-, CD8+/PD-L1-).

**Figure 4:**
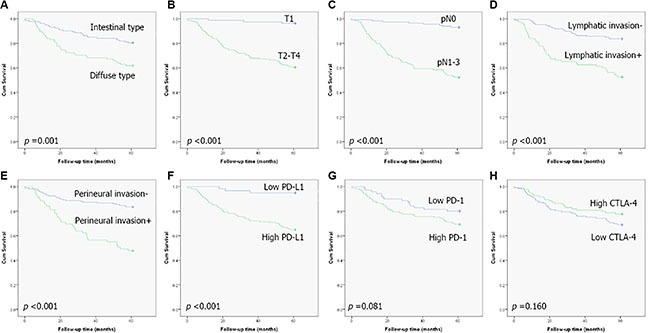
Kaplan-Meier plots of five-year survival for patients with high CD8+ tumor-infiltrating lymphocytes: (A) Lauren classification, (B) early versus advanced stage, (C) lymph-node metastasis, (D) lymphatic invasion, (E) perineural invasion, (F) PD-L1 expression, (G) PD-1 expression, and (H) CTLA-4 expression

Next, we performed a subgroup analysis dividing the patients into two groups in terms of high CD8+ and low CD8+ TILs. In the high CD8+ TIL group, high PD-L1 expression was significantly related to lower five-year OS in univariate analysis. (Figure 4) Multivariate analyses using Cox proportional hazard regression also revealed the independent prognostic value of PD-L1 expression (Table 4). In the CD8 low-expression group, a significant adverse survival effect was also seen for the high PD-L1 expression group in a univariate analysis (*p* value < 0. 001) but Cox multivariate analyses revealed no statistical significance.

**Table 4:** Multivariate analyses of variables in relation to five-year overall survival of patients with high CD8+ tumor-infiltrating lymphocytes

Variables	Parameter	Hazard ratio (95% CI)	*p*-value
Lauren classification	Diffuse vs. intestinal	1. 102 (0. 647−1. 878)	0. 72
Tumor stage	pT2-4 vs. pT1	0. 282 (0. 081−0. 986)	0. 047^*^
Lymph node metastasis	Present vs. absent	0. 285 (0. 125−0. 651)	0. 003^*^
Lymphatic invasion	Present vs. absent	0. 633 (0. 367−1. 093)	0. 101
Perineural invasion	Present vs. absent	0. 602 (0. 355−1. 022)	0. 06
PD-L1	High vs. low	0. 045 (0. 089−0. 974)	0. 045^*^

* Statistically significant *p*-values are indicated in boldface type.

Abbreviations: CI, confidence interval.

## DISCUSSION

This study used IHC staining to assess the prognostic significance of PD-1, PD-L1, and CTLA-4 in gastric adenocarcinoma samples.

We examined PD-L1 and CTLA-4 expression in tumor cells and CD8 and PD-1 expression in TILs in 464 cases of primary operable gastric adenocarcinoma using IHC analysis. PD-L1 and PD-1 expression was significantly correlated with several adverse prognostic pathologic factors, including higher T-stage, diffuse type of Lauren histologic type, and lymphatic invasion.

PD-L1 and PD-1 expression was also significantly correlated with high CD8+ TILs. PD-1 and PD-L1 expression showed a statistically significant correlation with each other.

Previously published results explored different tumor types with controversial or even directly conflicting results regarding the prognostic role of PD-1/PD-L1 expression. Although mainly poor treatment or survival outcomes associated with high PD-1/PD-L1 expression have been reported, a favorable prognostic value of this expression profile was reported in association with HPV-positive head-and-neck cancer patients and for some hematologic tumors [14, 15].

In a previous study of gastric cancer, high expression of PD-L1 in tumor cells was found to be significantly correlated with a worse prognosis [11]. Herein, our complete case analysis revealed that high PD-L1 and PD-1 expression had an adverse effect on five-year OS in univariate analyses. However, multivariate analyses failed to find statistically significant survival differences based on expression. We expected to find differences between patient subgroups; thus, we performed subgroup analyses by dividing patients into low and high CD8+ TIL groups and evaluating the prognostic differences in PD-1/PD-L1 expression between these groups. In our analyses, PD-L1 was a significant prognostic factor in the high CD8+ TIL group only.

According to a phase-2 colon-cancer clinical trial, tumors with mismatch-repair deficiency had better treatment outcomes than tumors without mismatch-repair deficiency after administration of pembrolizumab, an anti-PD-1 immune checkpoint inhibitor [16]. The authors suggest these results are due to a mutation-associated neoantigen, resulting in dense lymphocytic infiltration and a Th1-associated cytokine-rich environment of a mismatch-repair deficient tumor. In lung cancer cases, based on a multicenter phase-I clinical trial, the response of anti-PD-1 antibodies was correlated with higher PD-L1 tumor expression in advanced non-small-cell lung cancer patients [17]. However there is still no consensus about how to measure PD-L1 expression level and lack of PD-L1 expression does not exclude a potential response to anti-PD-1 antibodies. Many clinical trials have been conducted with gastric cancer patients. For example, a phase-lb trial for pembrolizumab, a monoclonal antibody of PD-1, is ongoing and there are not yet results indicating which patient subgroups may best benefit from checkpoint blockade immunotherapy [18]. The result of this study may help us better determine which patients are more likely to respond to immune checkpoint inhibitors in gastric cancer. Further research and clinical trials are needed to further evaluate the clinical usefulness of our findings.

The effect on CD8+ T-cell function through the PD-1/PD-L1 pathway remains to be clarified and our study indicated that there are no significant correlations between CD8+ TILs and five-year survival rates. The prognostic significance of CD8+ TIL for cancer is controversial. High CD8+ TIL levels are thought to be a good predictor of patient survival for a diverse set of human cancers, including gastric and ovarian cancer [19, 20]. However, adverse prognostic effects were also reported for ovarian cancer patients [9].

The value of anti-CTLA-4 monotherapy has been evaluated for many human cancers with varying results; it is likely to be particularly effective for intrinsically immunogenic tumors with a lower tumor burden [21]. In addition to T-cells, CTLA-4 expression is known to be present in a variety of cell types, including tumor cells, but the functional pathway is still unknown [5, 22, 23]. In breast cancer cases, CTLA-4 expression was associated with axillary lymph-node metastases and higher tumor stages [23]. Conversely in this study, CTLA-4 expression was correlated with lower T-stage, well-differentiated histologic grade, and intestinal Lauren histologic type. Ultimately, the prognostic role of CTLA-4 was not found to be statistically significant.

Our study has several potential limitations. We performed digital image analyses for our assessment of PD-1 and CD8 IHC outcomes to eliminate inter-observer variation, which could be problematic for obtaining accurate positive cell counts and intensity levels. However for CTLA-4 and PD-L1, we performed visual pathologic evaluations instead of digital-image analyses because of the inaccuracies in recognizing positive cells in digital image analyses due to diffuse staining patterns. Also, the proportions of tumor and stroma were different between all core and stromal cells were not completely excluded from counting during digital analysis.

In conclusion, we found statistically significant correlations between PD-1, PD-L1, and CD8+ TILs in gastric adenocarcinoma patients and revealed the prognostic value of PD-L1 expression in patients with high CD8+ TIL levels. More research using large randomized samples or independently validated cohorts are required for practical application.

## MATERIALS AND METHODS

### Patients and clinicopathologic data

In total, 464 formalin-fixed paraffin-embedded (FFPE) gastric adenocarcinoma tissue samples from patients who underwent surgical treatment at Korea University Guro Hospital from 2002 to 2005 were evaluated. Clinical data, including age, sex, disease specific survival, metastasis, and recurrence, were obtained through chart review. Pathologic data, including TNM stage, histological grade, Lauren classification, and presence of perivascular or perineural invasion were evaluated by slide review. Tumor staging was assessed according to the seventh edition of the American Joint Committee on Cancer (AJCC) [24]. This study was approved by the Institutional Review Board of Korea University Guro Hospital (KUGH12225).

### Tissue microarray block construction and immunohistochemical staining

Tissue microarray (TMA) blocks were constructed using tissue cores with 0. 2-mm diameters punched from the representative tumor area of the donor block. The TMA blocks were paired with normal-tissue controls. Serial 4-μm-thick sections were cut from the TMA block and mounted on electrostatic slides for IHC staining. IHC staining was performed using a standard streptavidin-biotin-peroxidase complex method. After deparaffinization with xylene, the tissues were rehydrated through a graded alcohol series and treated with 3% hydrogen peroxide for 20 minutes for endogenous peroxidase blocking. Next, antigen retrieval was done using 10mM citrate buffer (pH 6. 0) for 20 minutes. A BOND-MAX autostainer (Leica, Wetzlar, Germany) was used for IHC studies.

### Analysis of immunohistochemical staining

IHC staining revealed that PD-1 and PD-L1 had a membranous expression pattern, while CTLA-4 had a cytoplasmic expression pattern. CD8 had both a cytoplasmic and membranous expression pattern. For assessment of CTLA-4 and PD-L1 expression in tumor cells, IHC results were assessed and graded on a four-tier scale based on the intensity of positive cells (0, negative; 1, weak; 2, moderate; and 3, strong) by two experienced pathologists (KBH and CHY) who were blinded to all patient clinical data. Grades 0 and 1 were defined as low expression and grades 2 and 3 were defined as high expression. For evaluation of CD8 and PD-1 expression in T-lymphocytes, digital image analyses were done. For digital image analyses, whole-slide scans were done using a slide scanner (ScanScope CS, Aperio, CA, USA) and an image analysis program (Aperio ScanScope™, CA, USA). Using the image analysis program, the IHC intensity score was classified on a 4-tier system (0, negative; 1, weak; 2, moderate; and 3, strong). A score of 2 and 3 was considered positive. A median positive cell count of all tissue cores was used as a cutoff point and we stratified patients into two groups for each antibody. The antibodies used for IHC were: anti-PD-L1 antibody (1:100, rabbit polyclonal; Abcam, Cambridge, MA, USA), anti-PD-1 antibody (1:100, clone NAT105, mouse monoclonal; Abcam), anti-CD8 antibody (1:200, clone 4B11, mouse monoclonal; Leica Microsystems, Wetzlar, Germany), and anti-CTLA-4 antibody (1:50, clone F-8, mouse monoclonal; Santa Cruz Biotechnology, Dallas, TX, USA).

### Statistical analyses

Relationships between clinicopathologic factors and IHC results were sought using the chi-squared test or Fisher's exact test for categorical variables, and the Mann-Whitney test or Student's *t-test* for continuous variables. Five-year overall survival (OS) was used as the clinical outcome. The Kaplan-Meier method and the log-rank test were used to compare survival curves. For factors identified as statistically significant in univariate analyses, multivariate survival analyses were conducted to determine outcomes based on predictors. Multivariate analyses were performed with the Cox proportional hazards regression model for adjusting the hazard ratio of the IHC profile with established clinicopathologic factors. In all statistical analyses, significance was defined as *p* < 0. 05 (two-sided). All statistical analyses were performed using SPSS version 20 (IBM, Chicago, IL, USA).
